# Preserving US microbe collections sparks future discoveries

**DOI:** 10.1111/jam.14525

**Published:** 2019-12-12

**Authors:** K. Boundy‐Mills, K. McCluskey, P. Elia, J.A. Glaeser, D.L. Lindner, D.R. Nobles, J. Normanly, F.M. Ochoa‐Corona, J.A. Scott, T.J. Ward, K.M. Webb, K. Webster, J.E. Wertz

**Affiliations:** ^1^ Phaff Yeast Culture Collection Food Science and Technology University of California Davis Davis CA USA; ^2^ Department of Plant Pathology Fungal Genetics Stock Center Kansas State University Manhattan KS USA; ^3^ Soybean Genomics and Improvement Laboratory USDA‐ARS Rhizobium Germplasm Resource Collection Beltsville MD USA; ^4^ Center for Forest Mycology Research USDA‐Forest Service Northern Research Station Madison WI USA; ^5^ UTEX Culture Collection of Algae The University of Texas at Austin Austin TX USA; ^6^ Department of Biochemistry and Molecular Biology University of Massachusetts Amherst MA USA; ^7^ National Institute for Microbial Forensics & Food and Agricultural Biosecurity Oklahoma State University Stillwater OK USA; ^8^ Dalla Lana School of Public Health University of Toronto Toronto ON Canada; ^9^ USDA‐Agricultural Research Service Peoria IL USA; ^10^ Soil Management and Sugar Beet Research Unit USDA‐ARS Fort Collins CO USA; ^11^ Institute of Applied Life Sciences University of Massachusetts Amherst MA USA; ^12^ E. coli Genetic Stock Center Department of Molecular, Cellular and Developmental Biology Yale University New Haven CT USA; ^13^ Present address: Bolt Threads Inc. 5858 Horton Ave Emeryville CA 94608 USA

**Keywords:** algae, biotechnology, diversity, environmental mycology, fungi

## Abstract

Collections of micro‐organisms are a crucial element of life science research infrastructure but are vulnerable to loss and damage caused by natural or man‐made disasters, the untimely death or retirement of personnel, or the loss of research funding. Preservation of biological collections has risen in priority due to a new appreciation for discoveries linked to preserved specimens, emerging hurdles to international collecting and decreased funding for new collecting. While many historic collections have been lost, several have been preserved, some with dramatic rescue stories. Rescued microbes have been used for discoveries in areas of health, biotechnology and basic life science. Suggestions for long‐term planning for microbial stocks are listed, as well as inducements for long‐term preservation.

## Introduction

Repositories of our culture can be endangered by massive disturbances such as floods, wildfires or civil unrest. Recent examples: the Audubon Aquarium of the Americas in New Orleans lost most of their fish in the aftermath of Hurricane Katrina (Kaplan and Zarembo 2005), many priceless items were damaged or destroyed in the Notre Dame Cathedral fire of April 2019 (Wu *et al.* 2019), and over 200 years of artefacts were lost in the fall 2018 fire at the National Museum in Rio de Janeiro in Brazil (Gorman 2018). Another type of vulnerable ‘culture’ is particularly relevant to readers of this article: microbial cultures, which are often preserved in either public culture collections or in research collections. For instance in March 2019, there was a transformer explosion and fire in the Kline Biology Tower housing the *Escherichia coli* Genetic Stock Center (CGSC) at Yale University. Fortunately, all the freezers were evacuated from the building, and returned promptly with minimal loss of stock viability. However, this incident should serve as a wake‐up call to microbiologists and the impacted research community regarding the need to safeguard both private research collections and public microbial repositories, because important collections have indeed been lost. For example, the International Collection of Phytopathogenic Bacteria (ICPB) began at Cornell University in the 1920s, was transferred to the University of California Davis, then to the Plant Pathology department at the University of California Berkeley in the 1980s. The collection consisted of thousands of cryopreserved bacteria, representing all major bacterial pathogens of plants. When the department was disbanded in 1994, the collection was sent to USDA‐ARS in Beltsville (Milton Schroth, personal communication). One of the last publications by the recipient was in 2008, ironically a review of the importance of understanding emerging bacterial plant pathogens in the era of climate change (Schaad 2008). Current USDA employees were unable to locate the collection, except for a handful of strains that were deposited in the NRRL collection at USDA‐ARS in Peoria, IL (Bob Davis and Todd Ward, personal communication). All was not lost, however: the online American Type Culture Collection (ATCC) catalog contains 259 bacteria strains with ICPB strain numbers. As another example, eminent food microbiologist Dr. William Sandine (1928–2018) of Oregon State University greatly advanced the field of dairy starter cultures, and assembled a collection of thousands of food‐associated Gram positive bacteria and phages that infect them. When he retired in 1996, no replacement was hired, and his research laboratory was shut down. The phage collection was lost around 1995 when a cold room broke down (Bruce Geller, personal communication). A few strains that were lyophilized are still stored at room temperature in the OSU laboratory of Cindy Fisher, but they are not available to the public. A few dozen strains were incorporated into research collections of his laboratory alumni (Todd Klaenhammer, Jerri Bartholomew and Janine Trempy, personal communication). Recovery of this collection could benefit emerging research areas, such as the impact of the gut microbiome on human health.

Appreciation of the benefits of preserving collections, advance planning and support mechanisms can help avoid these types of losses. This article presents case studies and resources available to aid researchers in planning for the long‐term sustainability of microbial collections.

## Diversity of microbial collections

If you have worked with a microbe in a laboratory, chances are good that it came from a microbial culture collection. These collections include large public repositories that are accessible to the scientific research community, as well as collections that are not publicly accessible such as the research collection of one’s thesis advisor or an industrial research laboratory. They include genetic stock centres with thousands of genetic variants of the same species, and biodiversity collections which hold numerous species of wild‐type micro‐organisms. They can be held at universities, companies, government agencies and private institutions. Resources accessed by users include not only the microbes themselves, but also related materials including the data associated with the microbes, and the expertise of the curator. For this reason, the term Biological Resource Center (BRC) is often used to describe valuable collections (Smith *et al.* 2013), especially those that meet the guidelines detailed in the Organisation for Economic Cooperation and Development best practices guidelines (Organisation for Economic Cooperation and development 2007) or the ISO standards for biobanks (Allocca *et al.* 2017). Information about culture collections that have registered in the World Federation for Culture Collections is available online (http://www.wfcc.info). Examples of some prominent microbe collections in the United States, their major holdings and URLs are listed in Table 1 (McCluskey *et al.* 2016). Curators of these collections have interacted in recent years through the US Culture Collection Network (McCluskey *et al.* 2014a; McCluskey *et al.* 2014b; Boundy‐Mills *et al.* 2015; McCluskey *et al.* 2016; McClusskey *et al.* 2017b). Because this publication arose from discussions at a USCCN gathering, it focuses on US collections. Further information about collection rescue efforts in Europe and Asia.

**Table 1:** Back‐up Collections preserved at USDA ARS National Laboratory for Genetic Resource Preservation (NLGRP) in Fort Collins, Colorado

Taxa	Number of isolates	Home collection*	Location
Bacteria and fungi	>92 000	NRRL	USDA‐ARS, Peoria, IL
Penicillium	10	WRPIS	USDA Western Regional Plant Introduction Station, Pullman, WA
Phaff Yeast Culture Collection	3854	UC Davis	UC Davis, Davis, CA
Filamentous fungi	23 000	FGSC	Kansas State University, Manhattan, KS
Rhizobium	228		USDA‐ARS, Beltsville, MD
Tilletia	69		USDA‐ARS, Aberdeen, ID
ToMV/Fusarium	409/232	CPPSI	UC Davis, Davis, CA
Entomopathogen fungi	3497	ARSEF	USDA‐ARS, Biol Integ. Pest Mgt. Ithaca, NY
Rhizoctonia	210		USDA ARS, Soil Management and Sugarbeet Research Unit, Ft. Collins, CO
Bacillus	2569	BGSC	Ohio State University, Bacillus Genetic Stock Center, Columbus, OH
Pseudomonas	1765		USDA‐ARS, Salinas, CA
Verticillium Gibellulopsis	256		Bostock Lab, UC Davis, CA
Magnaporthe	882	NRRC	USDA‐ARS, Stuttgart, AR
*E. coli*	9700	CGSC	Yale University, E*. coli* Genetic Stock Center
Barnett Fungi	81		West Virginia University, Morgantown, WV
Algae	800	UTEX	The University of Texas at Austin, Austin TX

* Acronym definitions: Northern Regional Research Laboratory (NRRL); Western Regional Plant Introduction Station (WRPIS); Fungal Genetics Stock Center (FGSC); Collaboration for Plant Pathogen Strain Identification (CPPSI); ARS Collection of Entomopathogenic Fungi (ARSEF); Bacillus Genetic Stock Center (BGSC); Dale Bumpers National Rice Research Center (NRRC); E. coli Genetic Stock Center (CGSC); University of Texas Culture Collection of Algae (UTEX).

Further descriptions of various types of collections have been published, including yeast (Boundy‐Mills 2012; Boundy‐Mills *et al.* 2016; Groenewald *et al.* 2017), algae (Brand *et al.* 2013) and fungi (Smith 2004; Wiest *et al.* 2012). These repositories have preserved a broad range of organisms, facilitating future discoveries that could not have been envisioned when the organisms were preserved.

Major public repositories such as the ATCC have grown through deposits ranging from a single culture cited in a publication or patent to large sets deposited by retiring professors. Analogous to FAIR (Findable, Accessible, Interoperable and Reusable) guiding principles for scientific data, and to open access journals and open source software, Becker *et al. *(2019) recently proposed that scientific materials including microbes and genetic materials should be made more freely available to the research community. Preservation of micro‐organisms continues to be important to ensure that research can be replicated, to allow future discoveries, and to avoid the costs of re‐isolating microbes. In some cases, re‐isolation is no longer possible due to habitat destruction or other factors. Mutagen‐generated classical genetic mutant strains are impossible to replicate.

New obstacles to building collections include the Nagoya Protocol. Since 2014, countries have been enacting legislation to ensure that the country of origin receives some benefits from commercialization of their biodiversity, which has resulted in new hurdles for isolating microbes from outside the United States (Kariyawasam and Tsai 2018). It is now extremely difficult to build a geographically diverse microbial collection. This issue has risen in importance since Nagoya Protocol legislation impacted research use microbes of international origin (McCluskey *et al.* 2017a; McCluskey *et al.* 2018; Reichman 2018; Kariyawasam and Tsai 2018).

Major public repositories are vulnerable to major disturbances such as loss of power, fires, floods and civil unrest. Research collections are particularly vulnerable to loss when a professor retires, dies or moves on to a different area of inquiry. Similarly, formal collections can become at risk when funding lapses, or institutional or funding agency priorities change. Because collections have become harder to build, preservation of existing collections has risen in priority. Institutions, researchers, funding agencies and journal editors are encouraged to plan ahead for preservation of organisms that are collected or generated.

## Off‐site backup of microbial culture collections

Culture collections are vulnerable to many dangers. For example in the 1990s an agricultural agency *Rhizobium* collection in Iraq was destroyed during the second Gulf War. Fortunately, the USDA‐ARS *Rhizobium* collection in Beltsville, Maryland was able to provide over 200 strains of alfalfa‐related bacteria that were indigenous to Iraq (Patrick Elia, personal communication). While the generosity of other collection curators can aid recovery after this type of catastrophic loss, off‐site backup of collections can even more effectively mitigate such disasters. Most collections hold large numbers of strains not preserved in any other public collection.

**Figure 1:**
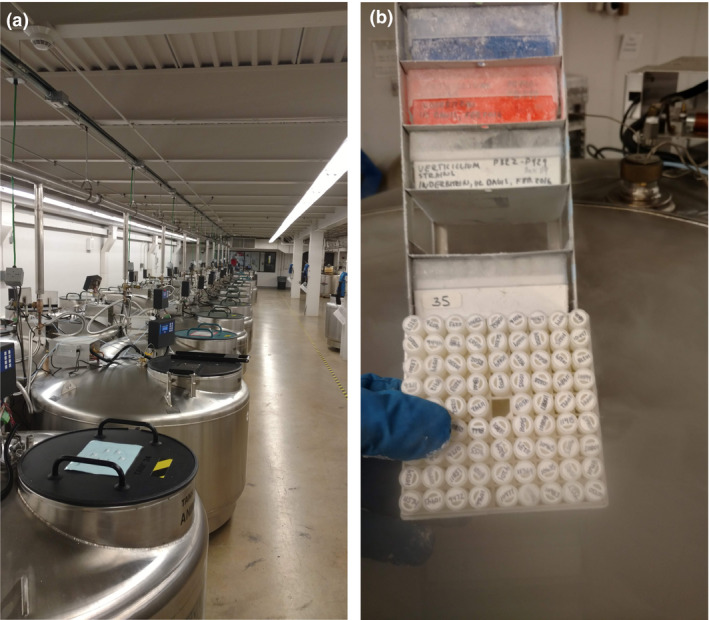
Liquid nitrogen tanks and vials of microbes, stored at the USDA‐ARS National Laboratory for Genetic Resource Preservation (NLGRP), Fort Collins, Colorado. (a) Liquid nitrogen tanks. (b) Vials of specimens cryopreserved in liquid nitrogen, vapour phase. [Colour figure can be viewed at wileyonlinelibrary.com]

Because collections are vulnerable to catastrophic loss, it is considered a fundamental best practice to preserve a copy of key resources at a secure off‐site location. Large, publicly funded health and defence‐related collections like the ATCC‐managed, National Institute of Allergy and Infectious Disease funded BEI Resources (www.beiresources) and related repositories include off‐site backup in their budget request (Simione and Cypess 2012). However, most living collections have historically relied upon *ad hoc* backups, leaving them vulnerable to catastrophe. To address this vulnerability, through collaborative engagement with the US Department of Agriculture National Plant Germplasm System and National Animal Germplasm Programs, many US microbial collections now store a backup copy of their holdings at the USDA‐ARS National Laboratory for Genetic Resources Preservation (NLGRP, Fig. 1) located in Ft. Collins CO (Table 1) (McCluskey *et al.* 2016). This program stores copies of USDA collections, such as the NRRL collection of the USDA‐ARS facility in Peoria, Illinois, and offered their services to the larger microbial resource communities over the last 10 years. Over a dozen public collections are now stored at the USDA under limited (5–10 years) Material Transfer Agreements. These collections are only stored as NLGRP does not distribute strains, except to return specimens to the original donor institution (or principal investigator) upon request. The 2018 California wildfires emphasized the need for backup storage, as they came within 30 miles of the University of California Davis campus. To mitigate this danger, a copy of the Phaff Yeast Culture Collection is cryopreserved at the NLGRP and would have been available to replace any specimens if so needed.

## Rescue of orphaned collections

A very common cause of loss of a private research collection of microbes is retirement of the researcher without a plan for the collection. The approach to preserving private research collections can include transfer to an existing collection or to an investigator at another institution, or by re‐staffing the same research area *in situ* and providing resources to sustain the collection. Some investigators prefer to disperse their research materials to colleagues or students who have helped assemble or utilized the collection in the past.

While many microbial collections are quietly destroyed, dispersed or lost, there are several stories of successful rescues of orphaned collections, dating back to the very first known microbial collection, that of Frantisek Král at the German University of Prague (Smith *et al.* 2014). His collection of fungi was transferred to the University of Vienna upon his death in 1911, then to Loyola University in the 1930s, then selected strains were transferred to ATCC (Uruburu 2003). In the early 1990s, CIAT transferred their entire Bradyrhizobium collection to the USDA‐ARS Rhizobium collection in Beltsville, MD. Rescued collections comprise cyanobacteria, algae, filamentous‐fungi, bacteria, yeast, chytrids and plant cell cultures. Many of these collections were transferred upon the retirement of the collection owners. Most transfers were accomplished without external support. Since 2014, funding for two microbial collection transfers was provided by the Collections in Support of Biological Research (CSBR) program of the National Science Foundation. The CSBR program recently funded transfer of 6000 plant‐associated bacteria from the University of Hawaii to Agdia, Inc. in Indiana (NSF award 1561663), where it is being utilized in agricultural screening projects. Thousands of yeast stocks were transferred from the Syracuse University and Tennessee State University to the University of California Davis, where they were deposited in the Phaff Yeast Culture Collection (NSF award 1561580).

In addition to funding, the ability to preserve these highly valuable and well‐utilized resources depends on availability of sufficient capacity at collections with staff trained in handling and maintenance of the specific resource. Currently the preservation of biodiversity in culture collections is moving towards a ‘distinct but united’ model where accessibility of data and common standards unites institutions that are free to focus on organism‐specific issues.

### Fungal Genetics Stock Center

Over many years, the Fungal Genetics Stock Center (FGSC) has accepted entire collections such as that of Nobel prize‐winner E.L. Tatum (Barratt 1986), a set of *Allomyces* strains (Olson 1984), and the collection of D. D. Perkins (Turner *et al.* 2001). Subsets have been acquired as well, including diverse mating types in *Schizophyllum* (Raper and Fowler 2004) or genetic mutants of the mushroom *Coprinopsis* (Burns *et al.* 2010) that have synchronized meiosis, to insure that highly cited strains remain available (Table 1). Among these, materials from the dawn of the classical genetics era have been utilized to document that freeze‐dried spores of *Neurospora* can survive nearly 65 years (McCluskey 2000). Without long‐term stability of collections, these types of studies can only be approximated. Over its 60+ year history the FGSC has migrated multiple times, first from its original institution, Dartmouth, to Humboldt State University in 1975, and when each successive director retired, to the University of Kansas Medical Center in 1985, to the University of Missouri—Kansas City in 2004, and most recently in 2014 to Kansas State University.

### Phaff Yeast Culture Collection at UC Davis

**Figure 2:**
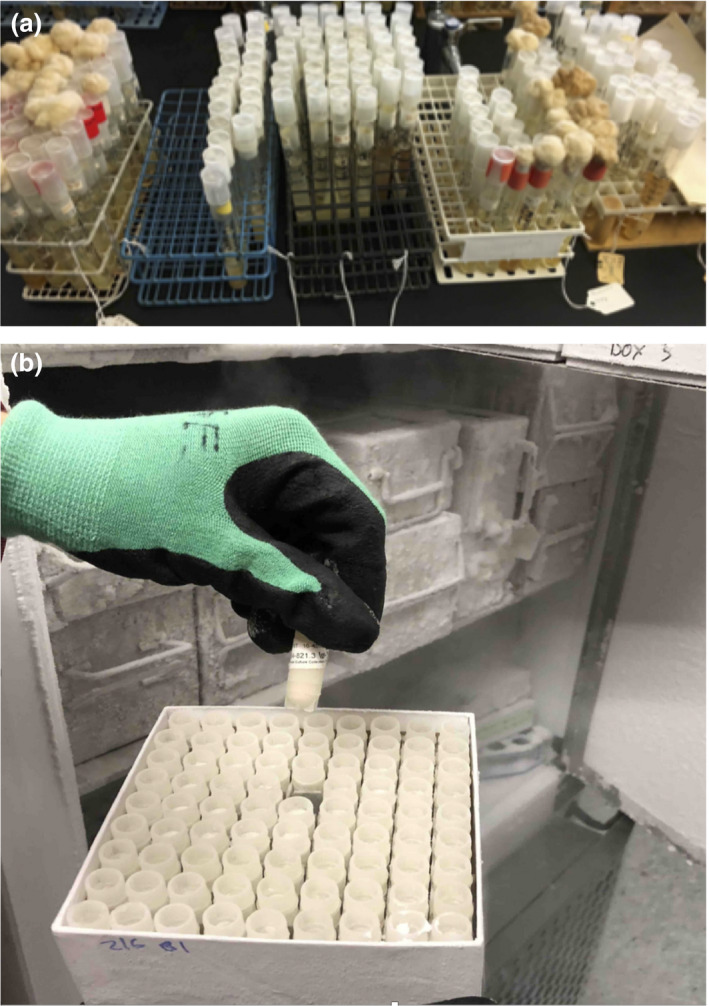
Rescued yeast specimens. (a) Rescued yeasts as preserved on agar slants by Starmer (viability limit 10–15 years). Note the cracked caps, yellowed cotton plugs and mineral oil overlay. (b) Cryopreserved yeasts in the Phaff Yeast Culture Collection (viable for decades). [Colour figure can be viewed at wileyonlinelibrary.com]

The Phaff Yeast Culture Collection at UC Davis is the fourth largest public collection of wild yeasts in the world (Boundy‐Mills *et al.* 2016). Originally established for understanding taxonomic biodiversity, the Phaff collection is used in‐house and by researchers around the world in diverse areas including taxonomy, functional genomics, biotechnology and food and beverage fermentations. For example the extensive biodiversity allowed discovery of yeasts that produce novel glycolipids (Cajka *et al.* 2016; Garay *et al.* 2017). Through establishment of sufficient capacity, and through funding from the US NSF (DBI 1561580) from 2016 to 2019, the Phaff collection was able to accession over 2600 unique strains when two researchers at other universities faced retirement (Table 1). Because the collection of Dr W. T. Starmer of Syracuse University was preserved as agar slants in glass tubes, topped with mineral oil, each glass tube had to be aseptically drained and sealed, packed in bubble wrap, then shipped from New York to California (Fig. 2a). Miraculously, only three tubes cracked during shipping. Over half of the stocks were viable, which was quite surprising as many were well over 20 years old but had an expected life of only 5–10 years. These yeasts are now cryopreserved, and expected to remain viable for decades (Fig. 2b).

### *E. coli* Genetic Stock Center

**Figure 3:**
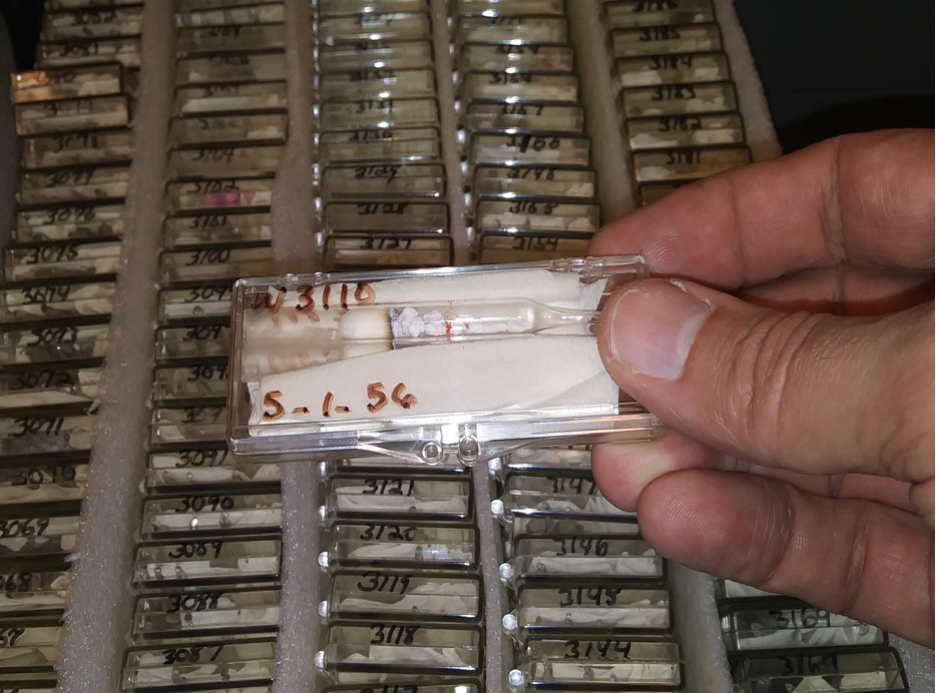
Lyopholized *Escherichia coli* from the collection of Nobel Prize winner Joshua Lederberg, preserved in the CGSC. [Colour figure can be viewed at wileyonlinelibrary.com]

Like many culture collections, the CGSC was created from the research collection of a single researcher: Ed Adelberg’s collection of ~6000 genetically characterized strains of *E. coli* at Yale University. Over the last 50 years, many small and several large collections from retiring researchers have been incorporated into the collection. Nobel Prize winner Joshua Lederberg was best known for discovery of transduction of DNA between bacterial strains through viral intermediaries. When he became president of Rockefeller University in 1978, he deposited his entire collection of 3516 lyophilized mutant bacterial strains into the CGSC (Fig. 3). When Frank Stahl, famous for discovering semiconservative DNA replication, retired from the University of Oregon, his entire collection of over 4000 strains was deposited in CGSC. In order to prevent the loss of important *E. coli* genetic resources, CGSC has expanded the collection to include *E. coli* bacteriophage. When Betty Kutter retired from Evergreen State College, CGSC acquired her collection of ~1100 genetically marked bacteriophage, which was itself composed of orphaned collections from several eminent researchers. Large collections are ironically in more danger of being lost when a researcher retires or unexpectedly dies, because they are simply too large to be taken in and properly managed in their entirety by a colleague or ex‐student.

### University of Texas Culture Collection of Algae

**Figure 4:**
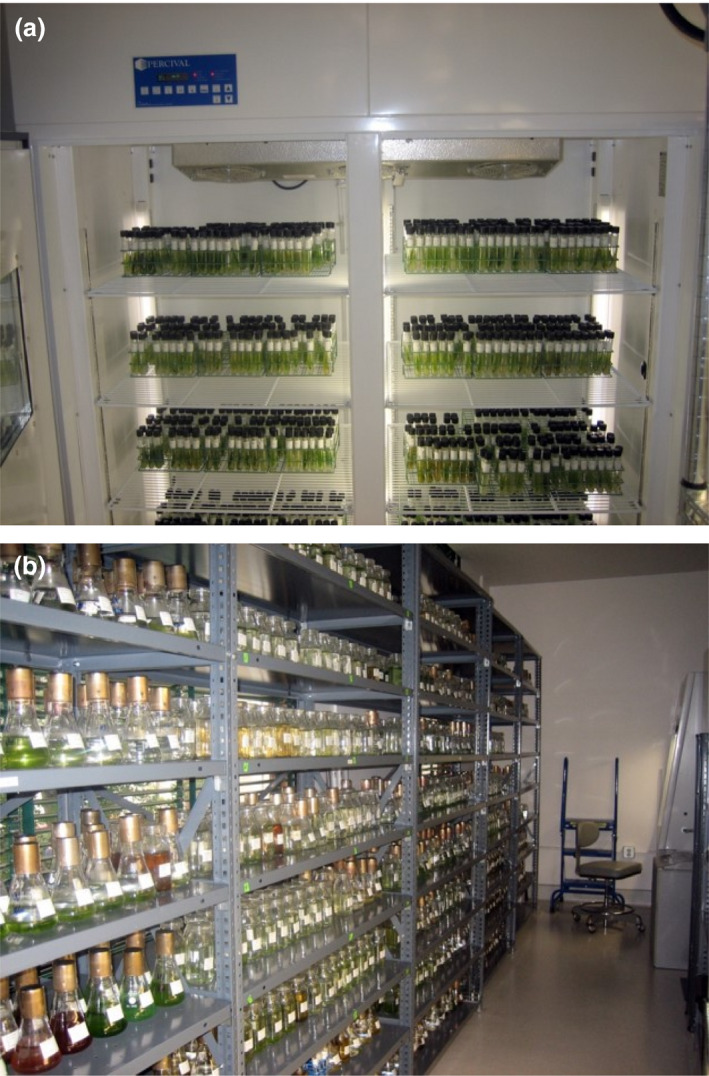
UTEX Culture Collection of Algae, Austin, Texas. (a) Active cultures of algae maintained as agar slants. (b) Active cultures of algae maintained as liquid cultures. [Colour figure can be viewed at wileyonlinelibrary.com]

Some algae can be cryopreserved without frequent attention. However, the majority of algal strains must be maintained as active cultures through perpetual serial transfers (Fig. 4). Transfer periods vary from weeks to months depending on the strain. Without the collection curator’s knowledge of numerous cultivation and preservation protocols, and appropriate space and equipment, many algae species would not be available to the research community (Brand *et al.* 2013). Over the past 15 years, the University of Texas Culture Collection of Algae (UTEX) Collection of Algae has rescued nearly 1000 algal strains from eight important research collections (Table 1) from environments like Antarctica, the Gobi Desert and a global snow algae collection. These collections are extremely diverse and include accessions of significant value to the fields of phylogenetics and systematics, cell biology, biotechnology, synthetic biology, ecology, environmental biology and astrobiology. All these collections were in danger of becoming orphaned due to death, retirement, loss of funding or loss of facilities.

### *E. coli* Reference Center

The *E. coli* Reference Center (ECRC) was established in 1967 at the Pennsylvania State University (PSU), and since the 1990s was an affiliate entity of the Animal Diagnostics Laboratory under the directorship of Dr. Chobi DebRoy. The ECRC houses approximately 88 000 strains, received from hundreds of geographical locations and diverse sources including human, animal and environmental. Most of the strains were typed for O and H antigen (Kauffmann 1946), and many for well‐known virulence genes (Scheinberg *et al.* 2017). The collection has also been used to generate sequence data for many of the O antigen gene clusters (DebRoy *et al.* 2011), for assessing genetic diversity within the species (Yin *et al.* 2013), and for surveillance studies (Magwedere *et al.* 2013). In January 2018, following Dr. DebRoy’s retirement, the ECRC was transferred to PSU’s Department of Food Science with funding provided by the College of Agricultural Sciences to aid in the transition. Projects primarily funded by the US Food and Drug Administration have generated genome sequences for over 4000 isolates during the past several years. At this time, nearly 1400 have been submitted to GenBank under Bioproject PRJNA357722, and all isolates will be available on the ECRC website in the near future (https://foodscience.psu.edu/research/centers/ecoli).

### Algal Research Collection

Hosted at the University of North Carolina Wilmington (UNCW), the Algal Research Collection (ARC) was saved by investment in the collection when the original investigator retired (Tomas 2012; Diaz *et al.* 2018). This collection is focused on algal species that generate toxins. Originally established at the Florida Marine Research Institute in 1987, its holdings began with a number of toxic dinoflagellates isolated from Florida local waters. Expanding to hold toxic taxa from other groups, the collection was transferred in 1999 from Florida to UNCW. Initially a private research collection, ARC became a service collection in September 2016.

### ARS Culture Collection (NRRL)

The NRRL was established in 1940 with mould cultures from the Thom and Church collection. Over the years, the NRRL expanded into a large biodiversity collection including bacteria, mould and yeast. As a public biodiversity collection with a broad mandate, NRRL has been able to acquire numerous research and institutional collections of microbes that might otherwise have been lost to science. These include the Blakeslee collection of Mucorales, the Mix collection of *Taphrina*, the US Army Quartermaster collection of filamentous fungi, the Smith collection of rhizobia, the International Streptomycete Project collection, the Waksman collection of Actinomycetales, and the Fell collection of marine yeasts. Currently, the NRRL is one of the largest public collections of micro‐organisms in the world, containing approximately 98 000 isolates of bacteria and fungi. The microbes maintained in the NRRL, including those obtained through acquisition of orphaned collections, are of critical importance to a wide variety of research projects around the world as evidenced by citation in more than 74 000 publications and patent applications indexed by Google Scholar. During the last 4 years, the ARS Culture Collection distributed over 23 000 microbial cultures in response to requests from scientists in 66 countries. However, reduced staffing and support for germplasm programs has made it nearly impossible for NRRL to absorb additional orphan collections in recent years.

### USDA‐ARS Rhizobium Germplasm Resource Collection

The USDA‐ARS Rhizobium Germplasm Collection is housed in the Soybean Genomics and Improvement Laboratory in Beltsville, MD. Some of its accessions were collected by USDA scientists over 100 years ago from test fields at the USDA’s Arlington Farm, where now the Headquarters of the US Department of Defense, the Pentagon, now stands. The collection has informally existed since 1913, but was officially established by an act of congress in 1975. It curates over 5000 accessions for over 450 legume species.

### UAMH Center for Global Microfungal Biodiversity

**Figure 5:**
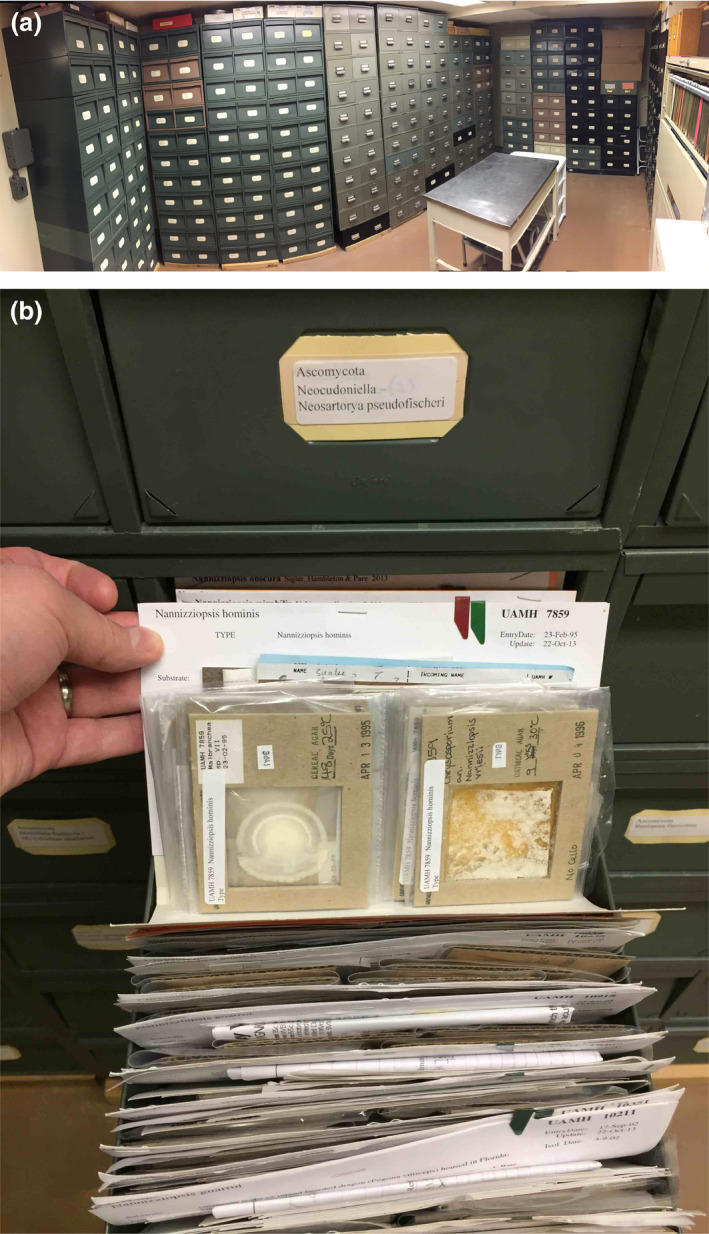
UAMH Center for Global Microfungal Diversity at the University of Toronto. (a) UAMH repository. (b) UAMH herbarium specimen. [Colour figure can be viewed at wileyonlinelibrary.com]

Another approach to preserving collections is the transfer of entire collections to a new institutional home. The former University of Alberta Microfungus Collection and Herbarium (UAMH) contains over 12 000 living strains of fungi preserved in multiple formats paired with dried culture herbarium specimens (Fig. 5). Many strains in the collection are highly cited in literature (Summerbell *et al.* 2018). In 2015, the entire collection was transferred from the University of Alberta to the University of Toronto. The transfer required heroic dedication of all parties involved against a short timeline of pending legislative changes that would have effectively precluded an inter‐provincial move. A defunct historic foundation was revived through legislative act in part to surmount inhibitory institutional policies; a broken drive shaft on a commercial moving truck necessitated midnight foraging for liquid nitrogen and electrical outlets; and a (survived) health emergency forced last‐minute restructuring to the delivery plan. Currently lacking institutional support or grant support, the UAMH collection relies on cost recovery from strain sales and goodwill. Because this collection holds both preserved colonies as well as living cultures, it represents a rare synergy between living and archival, preserved specimens.

### Chytrid fungi collection

Once‐private collections have been transferred into the public sphere. A collection of chytrid fungi was transferred from the University of Maine to the University of Michigan through support of the US NSF (DBI 1756202). These cultures will be made available to the public and managed as part of the University of Michigan Herbarium. Because the FGSC had maintained the Emerson Allomyces collection since 1995, these strains have been transferred to the new collection at Michigan (Table 1).

### Plant Cell Culture Library

Similarly, the Plant Cell Culture Library (PCCL), a well‐travelled collection of plant cell cultures, comprised of over 2000 different species of plants was established as a public resource at the University of Massachusetts (https://www.umass.edu/ials/pccl-database). Now residing in the University of Massachusetts Amherst (UMass), the PCCL was developed beginning in the 1990s by the company Phytera for discovery of anti‐infective agents (Mcalpine *et al.* 1999). The collection was subsequently acquired by Galileo Pharmaceuticals and then by a subsidiary of Monsanto Co., the Seminis Vegetable Seed Company. Monsanto donated the collection to UMass along with all rights to the gifted collection and with a grant from the US NSF (DBI 1561572), the living cultures that comprise the PCCL were shipped to UMass as callus (undifferentiated cells) growing on plates, or as cryopreserved cultures that are stored in liquid nitrogen Dewar tanks. Additional support from the UMass President’s Science and Technology Innovation Fund established the PCCL as a core facility within the UMass Institute of Applied Life Sciences. The PCCL is the world’s largest and most diverse collection of plant callus cell cultures, with over 1000 species of live plant cultures and an additional 1250 cryopreserved cell lines. Through this collaborative effort, these valuable research resources are available to the community for the first time.

### US Forest Service Center for Forest Mycology Research (CFMR)

The CFMR in Madison, Wisconsin contains approximately 20 000 isolates of 1600 forest‐associated fungal species, primarily basidiomycetes such as forest pathogens, wood‐decay and litter decay fungi. CFMR rescued 700 isolates of *Pseudogymnoascus*, which causes White Nose Syndrome of bats, isolated by the late professor Dr. Martha Christensen of the University of Wyoming. The collection has also preserved cultures collected by famous Forest Service and university mycologists, including O.K. Miller, H.H. Burdsall Jr., R.L. Gilbertson, L.O. Overholts, R.W. Davidson, as well as collections of two early female American mycologists, Frances Lombard and Mildred K. Nobles.

## Examples of recent discoveries made with rescued microbes

- New discoveries already made using yeasts rescued from the Starmer and Ganter yeast collections include six new species in the *Sporopachydermia cereana* complex, and a new strain of glycolipid‐secreting yeasts (unpublished data).
- Strains of *Pseudogymnoascus* fungi that cause White Nose Syndrome in bats that were rescued by CFMR were used to study the disease mechanism (Minnis and Lindner 2013; Palmer *et al.* 2014).
- The Plant Cell Culture Library has been screened for novel anti‐cancer activity (Addai *et al.* 2018).
- UAMH fungi have been used in recent studies of natural dyes and pigments (Robinson *et al.* 2018), and fungal infections in dogs (Townsell *et al.* 2018), insects (Sinia and Guzman‐Novoa 2018) and humans (Schwartz *et al.* 2018).
- Algae transferred to UTEX enabled their availability for such projects as constructing the Euglenid Tree of Life (Zakryś *et al.* 2017).

## Suggestions for researchers, funding agencies and journal editors

Significant effort goes into isolating or generating microbial strains. If preserved, countless discoveries will be made utilizing these microbes in the future. Therefore, researchers that collect or generate microbial strains are encouraged to think about the future of the organisms now. Suggestions for continued use of microbial resources have been developed (Organisation for Economic Cooperation and development 2007; Stackebrandt *et al.* 2015), and include:

- Organize and cull your collection routinely. Assign a unique identifier for each strain.
- Generate a database that contains the unique identifier, species, date isolated and origin (location and habitat), documentation including collecting permits, MAT and PIC documents and any characterization data such as genetic, physiological and biochemical data. (The date isolated and country of origin are particularly important for compliance with Nagoya Protocol legislation.)
- Learn about the Nagoya Protocol. Ensure that all international microbes are collected, used and distributed within the legal restrictions of the country of origin, including collecting permits, Mutually Agreed Terms (MAT) and Prior Informed Consent (PIC) documentation.
- Preserve microbes under conditions optimal for long‐term viability.
- Permanently label all preserved stocks with the assigned unique identifier.
- Share the database with at least one person in your organization, and at least one external person, who can connect it with the specimens if you are suddenly unavailable.
- If possible, preserve a copy of your collection off‐site.
- Deposit strains with research value in public repositories such as ATCC, NRRL or other collections listed in Tables 1 and 2.
- Identify an institution or laboratory with a long‐term mission of preservation and distribution that can absorb your collection.
- Explore funding opportunities for a collection transfer several years before you intend to retire.

**Table 2:** Collections rescued

Collection, Acronym (RRID)	Resource rescued	Number of isolates	Website
E. coli Genetic Stock Center, CGSC (RRID:SCR_002303)	E. coli K‐12 collection from Joshua Lederberg	3516	http://cgsc2.biology.yale.edu
	E. coli and lambda lysogen collection from Frank Stahl	4035	
	Evergreen Phage collection from Elizabeth Kutter	1100	
Fungal Genetics Stock Center, FGSC (RRID:SCR_008143)	*Coprinopsis cinereaus* from P. Pukkila (Burns *et al. * 2010)(25), UNC	164	http://www.fgsc.net/scripts/catalogDetails.asp?CatNum=161
	*Schizophyllum commune* from C. Raper (Raper and Fowler 2004), UVT	253	http://www.fgsc.net/scripts/catalogDetails.asp?CatNum=142
	*Neuropora sp* wildtype collection from D. Perkins (Turner *et al. * 2001), Stanford U	4789	http://www.fgsc.net/Neurospora/PerkinsWildCollectionatFGSC.xlsx
	*Ustilago maydis* from S. Leong/D. Perkins, U Wisconsin	119	http://www.fgsc.net/Umaydisddp.pdf
	*Allomyces *sp. from L. Lange (Emerson collection (Olson 1984))	254	http://www.fgsc.net/AllomycesStrainList.pdf
	*Neurospora crassa* mutants from EL Tatum (Barratt 1986)	1260	http://www.fgsc.net/Neurospora/tatumcollectlist.xls
	*Aspergillus nidulans* Temperature Sensitive‐Lethal mutant set (Harris *et al. * 1994)	1150	http://www.fgsc.net/tsmutant.html
Phaff Yeast Culture Collection, UCDFST (RRID:SCR_016781)	W. T. Starmer yeast collection, Syracuse University	1200	http://phaffcollection.ucdavis.edu/
	P. Ganter yeast collection, Tennessee State University	1400	http://phaffcollection.ucdavis.edu/
University of Texas Culture	Sigrid Berger, Dasycladales	25	https://utex.org/
Collection of Algae (UTEX) (RRID:SCR_016782)	Imre Friedmann, The Extreme Environment Collection	141	https://utex.org/pages/algal-collections
	Ron Hoham, Snow Algae	157	https://utex.org/pages/algal-collections
	David B. Czarnecki, Loras College Freshwater Diatom Culture Collection	459	https://utex.org/pages/algal-collections
	Juergen Polle, NAABB	30	https://utex.org/pages/algal-collections
	Willem Prud'homme van Reine, Sphacelariales	29	https://utex.org/
	Richard Triemer, Euglenid	80	https://utex.org/
	R. Malcolm Brown Jr., Siphonous Green Algae	37	https://utex.org/
University of Alberta Microfungus Collection and Herbarium, UAMH (RRID:SCR_016466)	UAMH collection of fungi, now at University of Toronto	12 400	https://www.uamh.ca/
Collection of Zoosporic Eufungi at the University of Michigan, CZEUM	Joyce Longcore’s Zoosporic fungi	580	https://czeum.herb.lsa.umich.edu/
PCCL (RRID:SCR_016784)	Plant Cell Culture Library	2000	http://www.umass.edu/ials/pccl-database
ARC (RRID:SCR_014942)	Algal Resources Collection	824	http://www.algalresourcescollection.com/
USDA ARS Rhizobium Germplasm	CIAT Bradyrhizobium Collection	2000	
Resource Collection	Peter Graham Collection	3055	

Researchers may be more prone to follow these suggestions if they become requirements for funding or publication. For example funding agencies are encouraged to urge researchers to include a ‘Specimen Management Plan’ with their funding proposals, similar to the Data Management Plan currently required for NSF proposals. Curators of professionally managed collections can provide guidance to PIs regarding what types and how many microbes they can absorb within a given time frame, what information they require for each microbe, and what the accession costs are. Many collections post deposit forms online. Because priorities shift when a project is completed, funding agencies should recommend that proposals include collection accession costs in the budget, and detailed plans to deposit significant organisms into a public collection in the project timeline.

Furthermore, journal editors are encouraged to require that authors deposit important research organisms into a public repository, much as they require deposit of DNA and protein sequences in public repositories. This is especially important for organisms used in genome sequencing projects. For example correlation of genomic information with physiological traits becomes impossible if the microbe was not preserved in a publicly available collection.

## Conclusions

Culture collections provide ‘life’ for life science research. Future discoveries in microbiology are enabled by continued access to authenticated resources which includes both micro‐organisms and associated data. Improved best practices are essential to ensure that collections that are otherwise orphaned or endangered are able to be maintained when faced with retirement or other transitions. Their continued maintenance through adequate funding and staffing is often a challenge. These challenges can be overcome through increased awareness of the conditions that make collections vulnerable, the consequences of loss of collections, and proactive steps by researchers, funding agencies and journal editors to ensure the viability of microbial collections.

Culture collections outside the United States are experiencing rapid growth due to enhanced institutional and government support, some prompted by Article 9 of the Convention on Biological Diversity. Non‐US collections are cautioned to plan for possible futures in which operational funding is reduced as it has been in the US.

## Conflict of Interest

The authors declare no conflict of interest.
